# Two Cases of Rare Pancreatic Malignancies

**DOI:** 10.1089/pancan.2019.0007

**Published:** 2019-07-23

**Authors:** Wissam Hanayneh, Hiral Parekh, Garrett Fitzpatrick, Michael Feely, Thomas J. George, Jason S. Starr

**Affiliations:** ^1^ Department of Medicine, Immunology and Laboratory Medicine, University of Florida, Gainesville, Florida.; ^2^ Department of Pathology, Immunology and Laboratory Medicine, University of Florida, Gainesville, Florida.; ^3^ Division of Hematology Oncology, Mayo Clinic, Jacksonville, Florida.

**Keywords:** clear cell, osteoclast-like giant cells, pancreatic cancer, undifferentiated

## Abstract

**Background:** Pancreatic adenocarcinoma remains one of the most lethal malignancies with little treatment advancements. Other less common pancreatic cancer histologies have different outcomes and disease course. In this article, we report two cases of rare pancreatic tumors.

**Presentation:** The first case is a 59-year old, who was undergoing surveillance of a known pancreatic cyst, which eventually enlarged. The mass was resected and pathology revealed undifferentiated carcinoma with osteoclast-like giant cells. The patient did not receive any adjuvant therapy and has had no recurrence. The second case is of a 60-year-old patient who presented with signs and symptoms of pancreatic insufficiency and was found to have clear cell adenocarcinoma of the pancreas. She received neoadjuvant chemoradiotherapy followed by surgical resection without complications.

**Conclusion:** Our article presents these rare malignancies, which had outcomes that are more encouraging than typical adenocarcinomas. Genomic sequencing can provide more insight into these tumors and potentially provide targets for therapy.

## Introduction

Pancreatic cancer remains one of the most lethal malignancies with a 5-year overall survival of 8% for all stages combined.^1^ Even more sobering is the fact that pancreatic ductal adenocarcinoma (PDAC) is projected to be second most common cause of cancer-related death in the United States by 2030.^2^

PDAC is the most common histology of pancreatic cancer (85–90%). However, several other histologies of pancreatic malignancies have been described. The World Health Organization (WHO) classifies pancreatic malignancies into epithelial and nonepithelial tumors. Histologically, epithelial subtypes include ductal, mucinous, serous, acinar, intraductal, pancreatoblastomas, and solid pseudo-papillary tumors. The nonepithelial tumors largely consist of neuroendocrine tumors. Despite the noted advancements of the field in the management of PDAC, unfortunately, there is a paucity of data on the rare histological subtypes of pancreatic carcinoma. The incidence, prognosis, and therapeutic outcomes of these rarer tumors are not well known and only a few such cases have been reported in the literature. In this report, we describe two cases of rare pancreatic tumors with the following histologies: undifferentiated carcinoma with osteoclast-like giant cells (UCOGC) and PDAC with clear cell features. In addition, we provide a review of the literature of these rare histological subtypes.

### Case 1: Undifferentiated carcinoma with osteoclast-like giant cells

**FIG. 1.**
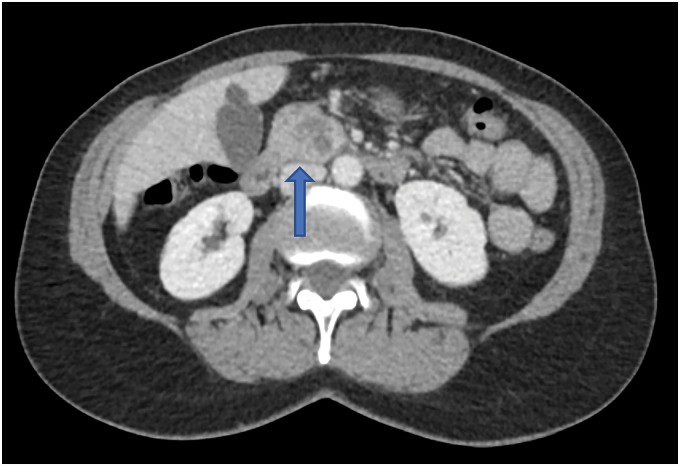
Axial CT scan of the abdomen in case 1 with intravenous contrast revealing a complex 2.3 cm pancreatic head lesion (arrow). CT, computed tomography.

A 59-year-old female with a history of papillary thyroid cancer (treated with radioactive iodine) was being followed with serial imaging for benign renal and hepatic cysts. At the time of her surveillance, magnetic resonance image (MRI), a simple 1.6 cm pancreatic cyst was noted in the pancreatic head, thought to be consistent with a benign cystadenoma. On repeat MRI 4 years later, the simple cyst had become complex and enlarged. Follow-up with a dedicated pancreatic triple phase computed tomography (CT) was performed, which revealed a 2.3 cm hypodense mass in the pancreatic head with a 4 mm enhancing component in the posterior aspect of the lesion with internal septations (Fig. 1). The patient then underwent endoscopic ultrasonography (EUS) with fine needle aspiration (FNA), which showed a high-grade undifferentiated carcinoma. The patient was completely asymptomatic without any laboratory abnormalities aside from a carcinoembryonic antigen (CEA) level of 4.6 ng/mL (normal ≤3 ng/mL). The patient's carbohydrate antigen 19-9 (CA 19-9) was within normal limits.

**FIG. 2.**
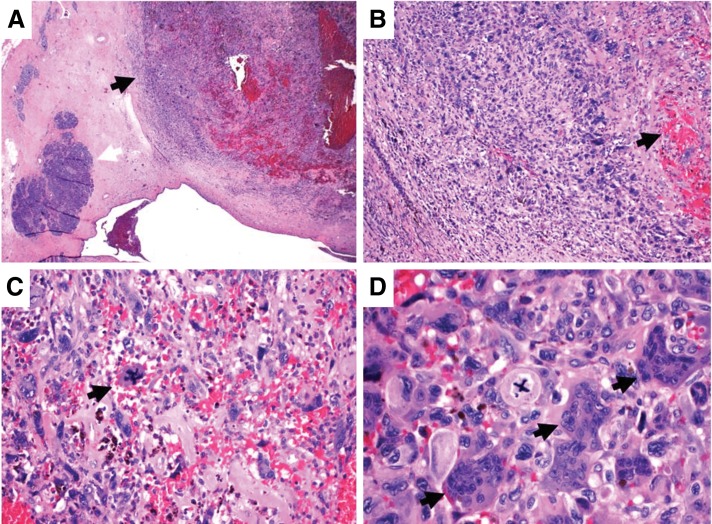
H&E-stained sections of UCOGC. **(A)** Low-power view demonstrating a fairly well-circumscribed tumor nodule surrounded by a rim of desmoplastic stroma (arrow), which abuts adjacent benign pancreas. Hemorrhage is observed within the tumor (arrow), 20× . **(B)** Central hemorrhage and necrosis give the appearance of a condensation of hyperchromatic nuclei toward the periphery of the nodule, 100× . **(C)** Multiple mitotic figures, including one bizarre pentapolar mitosis (arrow), are observed within a background of multinucleated giant cells and microscopic hemorrhage. Malignant cells demonstrate marked nuclear pleomorphism and hyperchromasia, 200× . **(D)** High-power view demonstrating an additional atypical mitotic figure surrounded by multinucleated giant cells (arrows) with a dense cytoplasm. Over 20 distinct nuclei can be observed within a single cell, 400× . H&E, hematoxylin and eosin; UCOGC, undifferentiated carcinoma with osteoclast-like giant cells.

Pancreaticoduodenectomy (Whipple procedure) was performed and the tumor was pathologically staged as IB (AJCC 8th edition, pT2N0M0). Macroscopically, the tumor was 2.3 cm in largest diameter. Microscopically, the tumor was well circumscribed with a pushing border, nodularity, and hemorrhage, and was composed of sheets of markedly pleomorphic mononuclear cells with interspersed non-neoplastic osteoclast-like (CD68+) multinucleated giant cells (Fig. 2A). Pleomorphic mononuclear cells stained positive for CK AE1/AE3 and CAM 5.2 by immunohistochemistry (IHC). Malignant cells were negative for S100, SOX-10, MelanA, and ERG. No conventional ductal adenocarcinoma component was identified. Furthermore, there were foci of hypercellular stroma with an epithelial-lined cystic structure suggestive of mucinous cystic neoplasm (MCN), which was now replaced by the malignant cells. This was consistent with UCOGC that developed within an MCN. Seventeen lymph nodes were retrieved and were all negative for malignant cells.

The patient did not receive adjuvant chemotherapy. Over 2 years after surgery, the patient has no evidence of recurrent local or distant disease with a CEA level of 2.8 ng/mL.

### Case 2: Clear cell adenocarcinoma

A 60-year-old female with a medical history significant for type 2 diabetes mellitus, hypertension, and hypothyroidism presented to her gastroenterologist after noting the gradual onset of epigastric abdominal pain radiating to her back. This was associated with a 30 lbs weight loss over a period of a few months. At that time, she reported that she found it more difficult to control her diabetes and required the addition of subcutaneous long-acting insulin to her regimen. Furthermore, she noted foul-smelling loose stools. Her gastroenterologist, concerned for symptoms of pancreatic malignancy and pancreatic insufficiency, performed an endoscopic retrograde cholangiopancreatogram, which showed a common bile duct obstruction. Cytologic brushings were obtained and a sphincterotomy was performed. The results of cytology were negative for malignancy. She continued to have worsening symptoms for another 3 months, so an EUS with FNA was eventually performed, which finally revealed the presence of cells consistent with pancreatic adenocarcinoma.

**FIG. 3.**
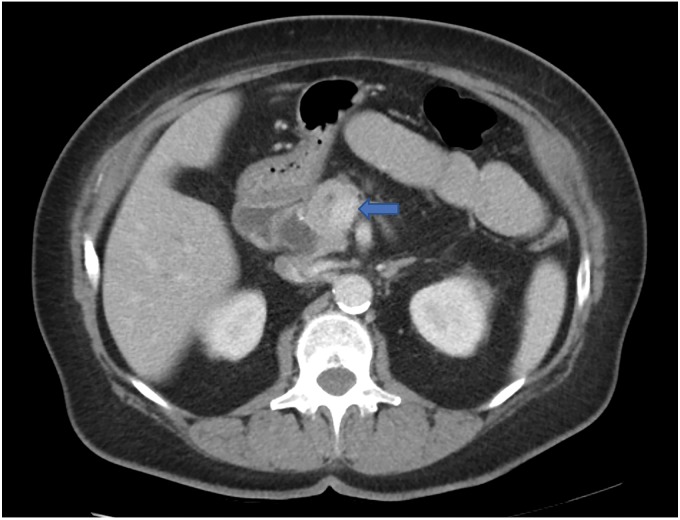
Axial CT scan of the abdomen in case 2 with intravenous contrast showing an ill-defined pancreatic head mass (arrow) that measures 3.6 × 3.6 cm. There is associated pancreatic ductal dilatation with abrupt cutoff at the level of the mass. The mass abuts the superior mesenteric vein and there is attenuation of the main portal vein and SMV at the confluence related to the mass. SMV, superior mesenteric vein.

At that time, her CA 19-9 was elevated to 170 U/mL. A CT scan described a pancreatic head mass measuring 3.6 cm × 3.6 cm, which did not extend inferior to the third part of the duodenum, but it seemed to abut the superior mesenteric vein and possibly the main portal vein as well (Fig. 3). There was dilation of the pancreatic, extrahepatic, and intrahepatic ducts. Given these findings, the mass was deemed to be borderline resectable. The decision was made to start neoadjuvant chemoradiotherapy with modified FOLFIRINOX regimen.

Following eight cycles of therapy, a repeat CT scan showed that the mass had shrunk to 2.2 cm × 2.7 cm and the patient was started on radiation therapy for a total of 28 fractions (5040 cGy) with concurrent capecitabine.

**FIG. 4.**
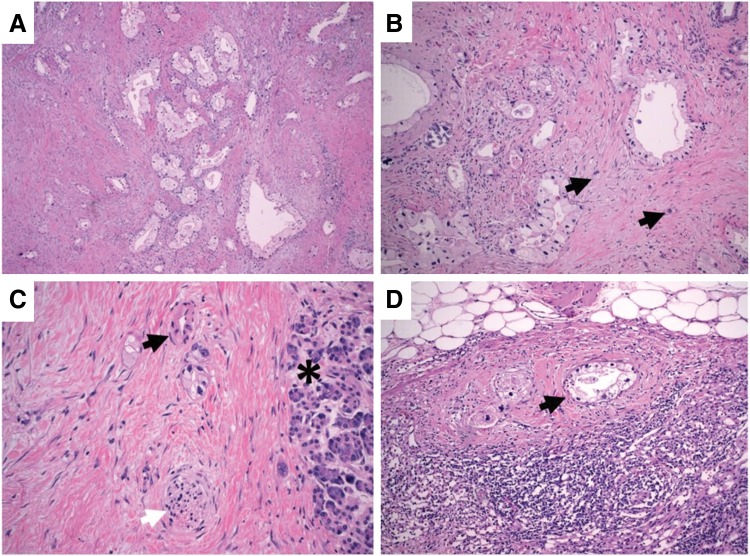
H&E-stained sections demonstrating clear cell features. **(A)** Low-power view demonstrating irregular malignant glands with surrounding desmoplastic tumor stroma. The normal lobular architecture of the pancreas is lost, 5× . **(B)** Angulated malignant glands are lined by epithelial cells with hyperchromatic basal to central nuclei of varying sizes and variable amounts of foamy to clear cytoplasm (arrows). Single malignant cells are present within the stroma, 100× . **(C)** Malignant cells are seen invading between an arteriole (black arrow), a nerve (white arrow), and adjacent benign pancreas (asterisk), 100× . **(D)** Similar histopathologic findings are observed in metastatic tumor glands seen in the subcapsular sinus of a peripancreatic lymph node (arrow), 100× .

Despite positive radiologic response, the patient's CA 19-9 continued to rise peaking at 422 U/mL upon completion of all neoadjuvant treatment. The patient successfully underwent a pancreaticoduodenectomy. Pathology revealed a 3.2 cm tumor with involvement of three of five regional lymph nodes. The tumor was staged as IIB (AJCC 8th edition, ypT2N1M0) with poor treatment response and positive uncinate margin. Interestingly, histology revealed PDAC with clear cell features (Fig. 4).

One of the patient's affected lymph nodes was sent for next-generation sequencing (FoundationOne^®^ CDx) testing, which revealed alterations in *KRAS* G12R (activating mutation), CDKN2A p16INK4a E120* (loss of function), and TP53 R273H (loss of function). Patient was placed on surveillance after the surgery. After 6 months, a CT scan showed multiple enlarging mesenteric and retroperitoneal lymph nodes in addition to multiple pulmonary nodules bilaterally, the largest of which measured 5 mm. Given the patient's CKDN2A loss-of-function mutation, the patient was enrolled in a phase I clinical trial utilizing ribociclib (inhibitor of cyclin D1/CDK4 and CDK6), in addition to gemcitabine.

## Discussion

### Undifferentiated carcinoma with osteoclast-like giant cells

This tumor was first described in 1968 by Rosai^3^ and was noted to contain two cell populations: osteoclast-like multinucleate giant cells and small mononuclear “stromal cells.” It was noted that these tumors were very similar to giant cell tumors (GCTs) of bone, thus termed “giant cell tumor of the pancreas.” Electron microscopy, however, revealed that both cell lines differed from GCTs of bone and had features of epithelial cell lines. Therefore, Rosai concluded that these tumors have an epithelial histogenesis. Later, multiple case reports disputed this conclusion when IHC showed staining for mesenchymal markers without features of epithelial cells.^4,5^ Early reports also distinguished two subtypes of GCTs: GCTs with osteoclast-like cells and pleomorphic GCTs^6^ to explain the disparity in the studies. However, it appears that most cases described thereafter have shown that all GCTs contain a mixture of both subtypes.^7^ In 2010, the WHO grouped them together under the term “undifferentiated carcinoma with osteoclast-like giant cells” (UCOGC).^8^

These tumors are composed of two distinct cell populations: undifferentiated mononuclear neoplastic cells, which stain positively for cytokeratins, and non-neoplastic multinucleated osteoclast-like giant cells, which are vimentin and CD68 positive. Histologic features, including a pushing border, tumor nodularity, hemorrhage, and peripheral concentration of giant cells, have been reported.^9–11^

The low proliferative index of the giant cells by Ki-67^12,13^ staining and their lack of driver mutations^14^ are highly suggestive of their benign nature.

Genetically, UCOGC of the pancreas resembles PDAC, with both entities harboring frequent *KRAS*,^10,13,15^ *TP53*, *CDKN2A*, and *SMAD4* mutations.^9^ The exact role of the osteoclast-like giant cells in UCOGC is not known, although they are thought to have phagocytic capabilities.^16^ The mechanism by which the giant cells are recruited to the tumor is also poorly understood. Further investigation is warranted as the osteoclastic giant cells could represent an upregulated immune response and contribute to our understanding of the biology of pancreatic UCOGC and other related tumors.

These tumors can be generally subdivided into two categories: pure osteoclast-like GCTs and those with a component of a more conventional neoplasm. Most often, the more conventional component is a PDAC associated with the GCT, although multiple cases in the setting of intraductal papillary mucinous neoplasm, MCN,^17,18^ high-grade pancreatic intraepithelial neoplasia (PanIN-3), and mucinous cystadenocarcinoma have been reported.^10^ The clinical course of these tumors has been variably reported. Early reports associated UCOGC with a dismal prognosis,^19^ although recent case series support a favorable prognosis with a 5-year overall survival rate of 59.1% versus 15.7% in PDAC following resection.^10,20^ The prognosis is especially favorable when tumors are of the pure UCOGC type and lack a conventional ductal adenocarcinoma component.^9,21^ As noted by Muraki et al., the correlation between increased survival and more predominant osteoclastic morphology raises the possibility that the presence of giant cells may represent a strong antitumor immune response to a conventional PDAC.^10^

The paucity of evidence leads to difficulty in determining the optimal multidisciplinary therapeutic approach to this rare malignancy. Surgical en-bloc resection is considered the first line of treatment for localized disease; however, the role of adjuvant chemotherapy is unclear. For the advanced stage and unresectable disease, given the epithelial origin of the mononuclear neoplastic cells, it may be reasonable to consider chemotherapy agents such as gemcitabine for palliation.^22^

Our case displays histologic, immunohistochemical, and clinical features consistent with UCOGC of the pancreas. After multidisciplinary management discussion, given the patient's early-stage disease and lack of data to support adjuvant therapy, the patient was placed on observation due to complete resection and remains disease free 2 years after surgery.

### Clear cell carcinoma

Clear cell morphology is seen in diverse solid malignancies. Metastatic renal clear cell carcinoma can present in the pancreas as late as a decade later.^23^ Furthermore, certain pancreatic tumors such as islet cell tumor, solid and cystic tumors,^24,25^ and perivascular epithelioid cell (sugar) tumors^26^ can have clear cell components to them. However, primary clear cell pancreatic carcinoma remains exceedingly rare. Literature review yielded around 10 case reports describing this rare histology,^27–37^ with the very first case described on autopsy in 1980,^33^ see Table 1 for a summary of all published English language case reports.

**Table 1.** A Summary of All Published English Language Case Reports

Case	Age, sex	Clinical presentation	Pathology	Staining profile	Mutations
Cubilla and Fitzgerald (1980)	Undetermined	Autopsy	NA	Positive for mucin	NA
Urbanski and Medline (1982)	57, Male	Abdominal pain and diarrhea	Giant cells with abundant eosinophilic cytoplasm admixed with malignant spindle cells interspersed between large cells with clear cytoplasm and hyperchromatic nuclei	Positive for PAS, PASD, and Alcian green. Negative for oil red 0	NA
Kanai et al. (1987)	71, Male	Abdominal and back pain	Cells with a clear cytoplasm in a solid and nested pattern	Positive for PAS and Alcian blue. Negative for sudan III	NA
Lüttges et al. (1998)	53, Male	Abdominal pain, weight loss, and jaundice	Large cells with clear cytoplasm	Positive for PASD, CK-7, 8, 18, 19, CAM5.2, and p53-DO7. Negative for vim, pancreatic stone protein, chromA, syn, serotonin, PP, alpha-HCG, substance P, and VIP	K-*ras* mutation codon 12
Ray et al. (2004)	75, Male	Incidental	Pleomorphic cells with clear cytoplasm and eccentric and pleomorphic nuclei	Positive for mucin (mucicarmine, PASD), CK-7, CAM5.2, CEA, NSE, A1AT Negative for vim, syn, chromA, p53, HMB-45, CD10	K-*ras* mutation codon 12
Sasaki et al. (2004)	61, Female	Epigastric pain and weight loss	Cells with abundant clear cytoplasm formed in nests and duct-like structures with fibrous stroma	Positive for PAS, PASD, CK-8, CK-19, A1AT, and CA 19-9 Negative for CEA, NSE, chromA, syn, insulin, glucagon, somatostatin, gastric, trypsin, and HMB-45	No K-*ras* mutation detected
Batoroev and Nguyen (2005)	60, Male	Epigastric discomfort	Malignant cells with abundant clear cytoplasm and pleomorphic nuclei	Positive for PAS, PASD, mucicarmine, and CEA Negative for vim	NA
Ray et al. (2004)	46, Male	LUQ pain and weight loss	Epithelioid cells with abundant clear cytoplasm	Positive for pancytokeratin and CK-7 Negative for PSA, TTF-1, thyroglobulin, vim, HMB-45, syn, and chromA	NA
Jamali et al. (2007)	75, Male	Abdominal distension, dyspepsia, jaundice, and weight loss	Clear cells with pleomorphic nuclei with raisinoid appearance. Squamous carcinoma with multinucleated giant cells. Large cells with eosinophilic cytoplasm forming adenocarcinoma	Positive for mucin and cytokeratins	NA
Lee et al. (2009)	66, Female	Epigastric pain and weight loss	Round to oval cells with clear cytoplasm and pleomorphic nuclei. Rhabdoid cellular features were seen	Positive for PAS, PASD, pancytokeratin, CK-7, CEA, and EMA Negative for CK-20, chrom, syn, SMA, and HMB-45	NA
Modi et al. (2014)	75, Female	Epigastric pain and weight loss	Pleomorphic cells with abundant clear cytoplasm	Positive for vim, CK-7, mucicarmine, PAS, PASD, CEA, and CA 19-9 Negative for AFP, CK-20, chrom, syn, HMB-45, Hep Par 1, and Glypican 3	NA
Sun et al. (2018)	64, Male	Epigastric pain and weight loss	Round to oval cells with abundant clear cytoplasm arranged in trabeculae, cords, and tubules. Pleomorphic nuclei present	Positive for CK-7 Negative for chromA, syn, and HNF-1β	NA
Current case	60, Female	Epigastric pain and weight loss	Malignant cells with pleomorphic nuclei and variable amounts of clear cytoplasm	NA	K-*ras* mutation codon 12

A1AT, alpha 1 antitrypsin; AFP, alpha fetoprotein; CA 19-9, carbohydrate antigen 19-9; CAM5.2, cytokeratin mouse antibody; CEA, carcinoembryonic antigen; chromA, chromogranin A; CK, cytokeratin; EMA, epithelial membrane antigen; HCG, human chorionic gonadotropin; HMB-45, human melanoma black 45; HNF-1β, hepatocyte nuclear factor-1β; NSE, neuron-specific enolase; PAS, periodic acid-Schiff; PASD, periodic acid-Schiff with diastase; PP, pancreatic polypeptide; PSA, prostate-specific antigen; SMA, smooth muscle actin; syn, synaptophysin; TTD-1, thyroid transcription factor 1; vim, vimentin; VIP, vasoactive intestinal peptide.

The tumor has been shown to arise in any part of the pancreas and the literature reports more cases in males. All previous cases reported elevation of CA 19-9 on presentation, indicating a ductal origin.

On IHC, clear cells are typically negative for acinar (trypsin and chymotrypsin) and neuroendocrine markers (synaptophysin and chromogranin,), but epithelial markers are usually positive, indicating a ductal origin. The similarity of IHC staining patterns between PDAC and clear cell carcinoma makes the distinction challenging and the diagnosis is predicated on the morphological appearance on microscopy. Kim et al.^28^ suggested the use of hepatocyte nuclear factor-1β as a marker to distinguish clear cell carcinomas from their more common ductal counterparts. Furthermore, stronger staining patterns on IHC may translate to worse prognosis.^38^ Perivascular epithelioid cell tumor, “sugar tumor,” is another pancreatic tumor with clear cell morphology; however, histologically, the cells are composed of epithelioid cells. IHC is usually negative for epithelial markers and for actin and melanogenesis-related marker (HMB-45), which differentiates it from clear cell carcinoma.^26^

Our case had a genetic alteration in the K-*ras* gene at codon 12 similar to two other cases in the literature.^29,35^

The optimal treatment and prognosis of these tumors are unknown. Multiple cases show the recurrence of the tumor in the form of liver metastasis after surgery.^29,38^ Surgical resection is offered in localized disease, while optimal systemic therapy is unknown in the setting of advanced unresectable disease. Two of the case reports have shown minimal response to 5-fluorouracil and gemcitabine chemotherapy.^27,38^

## Conclusion

This report serves to showcase two rare pancreatic malignancies that clinicians can encounter in their practice. Because of the rarity of these tumors, treatment approaches are not guided by clinical trials. Molecular characterization of these tumors with next-generation sequencing, whole-exome analysis, and evaluation of biomarkers for response to immunotherapy (i.e., PD-L1, MMR/MSI, and tumor mutational burden) could potentially give us a better understanding of these diseases and help guide treatment.

## Abbreviations Used

CA 19-9: carbohydrate antigen 19-9
CEA: carcinoembryonic antigen
CT: computed tomography
EUS: endoscopic ultrasonography
FNA: fine needle aspiration
GCT: giant cell tumor
IHC: immunohistochemistry
MCN: mucinous cystic neoplasm
MRI: magnetic resonance image
PDAC: pancreatic ductal adenocarcinoma
UCOGC: undifferentiated carcinoma with osteoclast-like giant cells
WHO: World Health Organization
